# Computed Tomography Yield and Predictors of Pulmonary Embolism in Mechanically Ventilated Patients—A Retrospective Study

**DOI:** 10.1111/aas.70216

**Published:** 2026-03-08

**Authors:** Erik Bruno, Matilda Kewenter, Angeliki Dimopoulou Creusen, Freyr Einarsson, Gaetano Perchiazzi, Kristina Svennerholm, Christian Rylander

**Affiliations:** ^1^ Department of Surgical Sciences Anaesthesiology and Intensive Care, Uppsala University and Uppsala University Hospital Uppsala Sweden; ^2^ Department of Surgical Sciences Radiology, Uppsala University and Uppsala University Hospital Uppsala Sweden; ^3^ Department of Anaesthesiology and Intensive Care Institute of Clinical Sciences, Sahlgrenska Academy, University of Gothenburg Gothenburg Sweden; ^4^ Department of Surgical Sciences, Hedenstierna Laboratory Uppsala University Uppsala Sweden

**Keywords:** computed tomography, critical care, ICU, intensive care, pulmonary embolism, risk factor

## Abstract

**Background:**

The reported prevalence of pulmonary embolism (PE) detected during intensive care varies substantially. Computed tomography pulmonary angiography (CTPA) is the diagnostic reference method, but assessing pre‐test probability and finding distinct indications for CTPA from clinical observations is difficult in mechanically ventilated critically ill patients.

**Aims:**

To describe the rate of positive diagnostic outcome (yield) from CTPA exams performed on PE suspicion and to identify predictors in the free text motivational reasons from the referring clinician and among selected patient variables during intensive care for other conditions.

**Methods:**

CTPA exams and reports in adult, mechanically ventilated patients referred from two intensive care units during a 5‐year period were compared to the radiology order content and to data collected from medical records. The association between a positive exam and five arbitrarily defined reasons for ordering the exam as well as selected patient variables was assessed using multivariable logistic regression.

**Results:**

Among 1113 thoracic computed tomography exams, 243 were CTPA ordered on clinical PE suspicion, yielding 52 (21% [95% CI: 17–26]) positives. Reasons “Elevated D‐dimer” and “Undefined clinical suspicion” were significantly associated with a positive exam. However, none of the selected patient variables was significantly associated with a CTPA finding of PE.

**Conclusion:**

The diagnostic yield of clinical CTPA exams for suspected PE in mechanically ventilated patients fell within a range commonly cited for suspected acute PE in emergency department populations, but its association with clinical signs described in the CTPA order and with selected patient variables was low.

**Editorial Comment:**

The report describes a single center experience, including 2 years of the COVID‐19 pandemic, with computed tomography pulmonary angiography performed to evaluate suspected pulmonary embolism in mechanically ventilated ICU cases. An elevated D‐dimer as well as high degree of clinical suspicion were associated with a positive pulmonary embolism finding on the radiological examination.

## 1 Introduction

Venous thromboembolism (VTE), including deep venous thrombosis (DVT) and pulmonary embolism (PE), is a well‐known complication in patients admitted to the intensive care unit (ICU) for other conditions [1]. During intensive care, risk from specific circumstances such as immobilization, sedation, mechanical ventilation, and pro‐thrombotic states is added to general risk factors for VTE [2]. The reported incidence of VTE varies substantially depending on the diagnostic method and population of critically ill patients studied [1]. The reference diagnostic test for PE is computed tomography pulmonary angiography (CTPA) using intravenous iodine contrast [3]. However, the exam may be withheld due to a perceived risk of contrast‐associated acute kidney injury and hazards associated with transporting critically ill patients to the radiology department [4, 5]. Furthermore, PE occurring in the ICU is often silent, with typical respiratory and circulatory failure ascribed to alternative diagnoses [6]. Clinical instruments developed for assessing pre‐test probability of acute PE are less useful in mechanically ventilated, critically ill patients [7]. This makes it uncertain on what grounds a CTPA exam for PE is ordered by intensive care clinicians. It is also sparsely described in the literature to which extent clinical variables are associated with PE during intensive care [8].

The aim of this retrospective study was to describe the diagnostic outcome and to identify predictors in the free‐text motivation from the referring clinician and among selected patient variables in mechanically ventilated patients referred to CTPA on clinical suspicion of PE during intensive care for other conditions.

## 2 Methods

This single‐center cohort study was approved by the Swedish Ethical Review Authority (DNr 2022‐03568‐01). Patient consent was waived due to the nature of the retrospective analysis of pseudonymized data. The study is reported according to the Strengthening the Reporting of Observational Studies in Epidemiology (STROBE) statement [9].

### 2.1 Patients and Settings

The study was conducted in the Uppsala University Hospital, an 800‐bed tertiary center for mid‐northern Sweden, serving a population of ~2 million. The study period lasted from August 1, 2016, to December 31, 2021. All adult (> 18 years), mechanically ventilated patients in the 8‐bed general ICU and the 8‐bed neurosurgical ICU who underwent a CTPA exam for clinically suspected PE were eligible. Patients with an inconclusive CTPA exam for any reason were excluded.

### 2.2 Data

The patients were identified through procedure codes in the local radiological archive from which CTPA exams, the radiology reports and text details of the orders were extracted. The presence of PE was established from the radiology reports, for which the standard operating procedure stated that findings must be assessed by two radiologists, of whom at least one is a consultant in radiology. The right‐to‐left ventricular transverse diameter (RV/LV) ratio was measured for the purpose of the study in axial sections on the CTPA images by one of the authors (MK) after saturated training by a consultant in radiology. Demography, medical history including past and present relevant ICD‐10 diagnosis, surgery, clinical, and laboratory parameters, and any dose of pharmacological thrombosis prophylaxis were collected from digital patient record systems. The physiological and laboratory parameters were taken from the last entry available before the CTPA exam except for creatinine which was sampled from three occasions: The last value before CTPA, the first value after CTPA, and the last value before discharge from the ICU. We recorded established risk factors for PE including a main ICU diagnosis of sepsis, polytrauma, isolated traumatic brain injury (TBI), surgery within 30 days, and COVID‐19 [10, 11, 12]. Admission to the neurosurgical ICU, a history of malignancy and the presence of thromboprophylaxis were also added to the analysis [13].

From the free text in the radiology orders, we identified thematic motivational reasons, used by the referring clinicians for motivation of PE suspicion, and arbitrarily grouped them into five categories: “Hypoxemia,” “Hypercapnia,” “Unstable circulation,” “Elevated D‐dimer,” and “Undefined clinical suspicion.” We did not use predefined operational definitions or threshold values but assessed the qualitative content as reflecting the clinical decisions to perform the exam. One CTPA order could contain more than one reason. Text referrals without motivation were not categorized.

### 2.3 Outcomes

Outcomes were defined as the crude proportion of positive CTPA exams (yield) and their association with five arbitrarily defined reasons for ordering the exam. Additionally, we examined the association between selected patient variables and a positive exam.

### 2.4 Statistical Analysis

For group statistics, continuous variables are presented as mean (SD) or median (first and third quartile; Q1:Q3) according to distribution and dichotomous variables are presented as numbers (%). Wilson exact score confidence intervals (CI) are given for the main outcome proportions. The Mann–Whitney *U*‐test or Fisher's exact test was used for respective comparisons between the groups. The association between the reasons given in the CTPA orders and a positive CTPA exam, expressed as the event odds ratio (OR) with corresponding 95% CI, was assessed using multivariable penalized logistic regression with Firth's correction, entering all categorized reasons into the model. To assess the association between selected patient variables and a positive CTPA exam, we applied first univariable, then multivariable logistic regression. Variables showing an OR above 1 and a *p* ≤ 0.20 in the univariable analysis and with less than 5% missing values were included in the multivariable model. Statistical analysis was performed using R version 4.5.2 (The R Foundation for Statistical Computing, Vienna, Austria), with penalized logistic regression implemented using the *logistf* package.

## 3 Results

**FIGURE 1:**
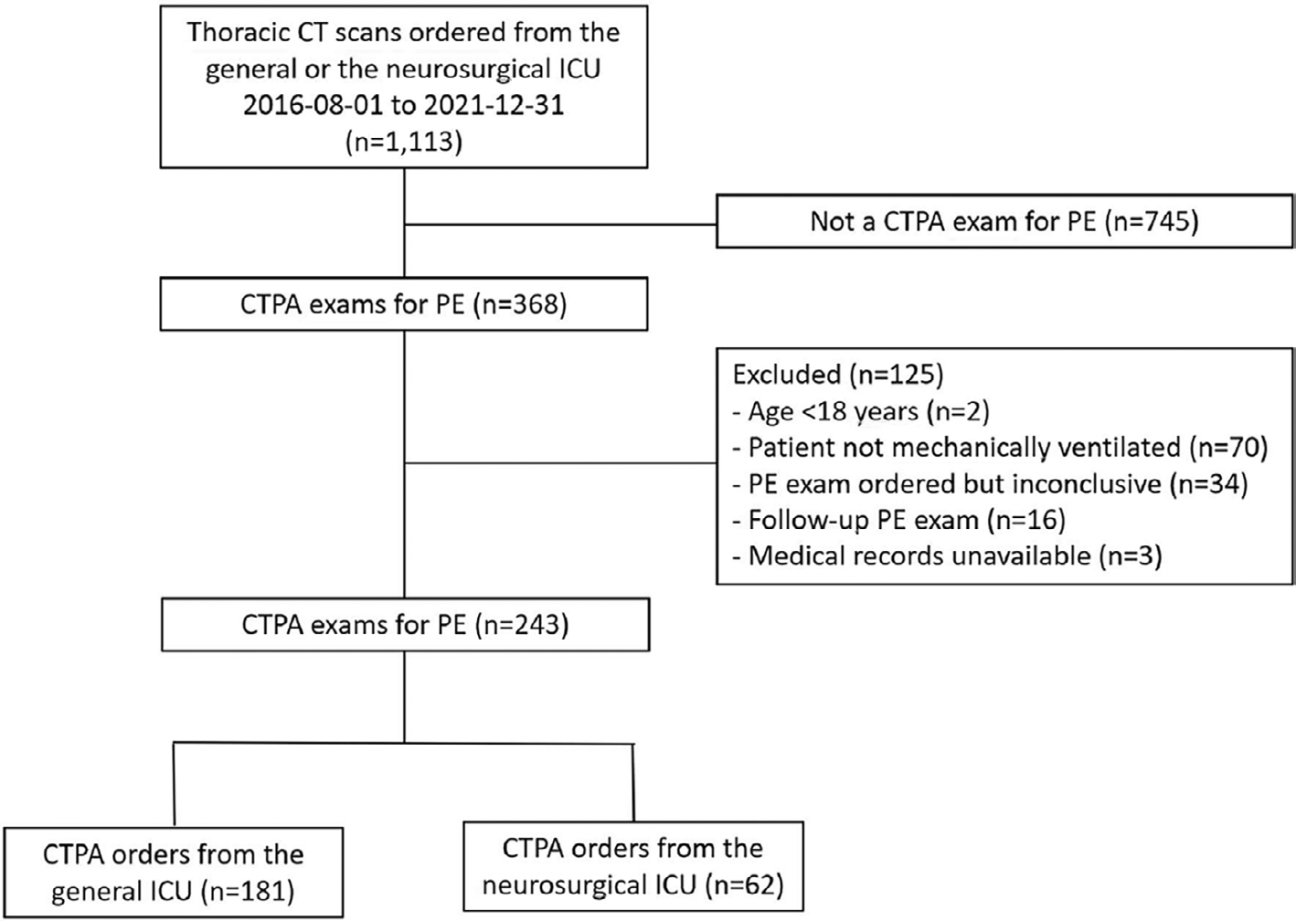
Flow chart.

During the 5‐year study period, there were 7498 patient admissions and 1113 computed tomography exams of the chest registered for the two ICUs. After exclusions, 243 adult patients with a mean (SD) age of 60 (15) years met criteria for a unique CTPA exam on PE suspicion (Figure 1). PE was found in 43/180 (24% [95% CI: 18–31]) men and in 9/63 (14% [95% CI: 7.7–25]) women, totaling 52/243 (21% [95% CI: 17–26]) patients. The emboli were located in the pulmonary artery bifurcation in 2 (3.8%), the central/lobar arteries in 17 (33%), the segmental arteries in 21 (40%), and the subsegmental arteries in 14 (27%) cases.

Patients with a positive finding on CTPA were younger and had a lower prevalence of heart failure and asthma. Thromboprophylaxis using low molecular weight heparin was administered to 36 (69%) of the patients with and to 128 (67%) of the patients without a positive exam for PE (Table 1). There was no non‐pharmacological thromboprophylaxis used alone. There was no difference between the groups as to creatinine levels before and after CTPA exams (Table 2), vital signs, or RV/LV ratio (Table 3).

**TABLE 1:** Clinical characteristics.

Variable	*Missing*	CTPA positive *n* = 52	*Missing*	CTPA negative *n* = 191	*p*
Demographics					
Age (years)	*0*	60 (46:68)	*0*	66 (54:73)	< 0.01
Female	*0*	9 (17)	*0*	54 (28)	0.15
Weight (kg)	*0*	88 (77:106)	*2*	87 (74:104)	0.39
BMI (kg/m^2^)	*1*	29 (25:33)	*10*	29 (25:34)	0.5
Co‐morbidities					
Hypertension	*0*	27 (52)	*0*	94 (49)	0.76
Heart failure	*0*	1 (1.9)	*0*	22 (12)	0.03
Cardiac arrhythmia	*0*	6 (12)	*0*	28 (15)	0.66
Ischemic heart disease	*0*	3 (5.8)	*0*	22 (12)	0.31
COPD	*0*	0	*0*	13 (6.8)	0.08
Asthma	*0*	1 (1.9)	*0*	30 (16)	< 0.05
Restrictive lung disease	*0*	0	*0*	0	—
Diabetes Type 1	*0*	0	*0*	2 (1.0)	1.00
Diabetes Type 2	*0*	14 (27)	*0*	35 (18)	0.18
Chronic renal failure	*0*	0	*0*	11 (5.8)	0.13
Acute on chronic renal failure	*0*	0	*0*	4 (2.1)	0.58
Acute kidney injury	*0*	3 (5.8)	*0*	15 (7.9)	0.77
Coagulation disorder	*0*	1 (1.9)	*0*	2 (1.0)	0.52
Previous DVT	*0*	0	*0*	6 (3.1)	0.35
Previous PE	*0*	1 (1.9)	*0*	5 (2.6)	1.00
Malignancy	*0*	6 (12)	*0*	37 (19)	0.22
Diagnosis at risk for VTE					
Sepsis	*0*	5 (9.6)	*0*	29 (15)	0.37
Polytrauma	*0*	3 (5.8)	*0*	8 (4.2)	0.71
Isolated TBI	*0*	6 (12)	*0*	16 (8.4)	0.58
COVID‐19	*0*	21 (40)	*0*	74 (39)	0.87
Surgery within 30 days	*0*	17 (33)	*0*	43 (23)	0.15
Thromboprophylaxis					
Low molec. weight heparin^a^	*0*	36 (69)	*0*	128 (67)	0.84
ICU stay					
Days in ICU in total	*0*	14 (9:22)	*0*	11 (5:21)	0.06
Days in ICU before CT	*0*	3 (1:10)	*0*	3 (1:8)	0.19
Survival					
Alive at ICU discharge	*0*	44 (85)	*0*	155 (81)	0.68
Alive 30 days after CTPA	*0*	42 (81)	*0*	136 (71)	0.15

*Note:* Values for continuous variables given as median (Q1:Q3) with *p*‐values referring to the Mann–Whitney *U*‐test. Values for dichotomous variables given as number (%) with *p*‐values referring to the Fischer's exact test. Italic figures indicate missing values.

Abbreviations: BMI, body mass index; COPD, chronic obstructive pulmonary disease; CTPA, computed tomography pulmonary angiography; DVT, deep venous thrombosis; ICU, intensive care unit; PE, pulmonary embolism; TBI, traumatic brain injury; VTE, venous thromboembolism.

^a^ Any dose prescribed at the time of the CTPA exam.

**TABLE 2:** Laboratory parameters before (and Creatinine after) the CTPA exam.

Parameter	*Missing*	CTPA positive *n* = 52	*Missing*	CTPA negative *n* = 191	*p*
pH	*1*	7.41 (7.37:7.45)	*3*	7.40 (7.34:7.45)	0.55
P_a_CO_2_ (kPa)	*1*	5.60 (5.10:6.50)	*3*	5.60 (5.15:6.40)	0.84
P_a_O_2_ (kPa)	*1*	10.6 (9.80:12.80)	*3*	10.80 (9.35:12.65)	0.62
Base excess (mmol/L)	*1*	2.8 (−0.1:4.1)	*3*	2.1 (−0.9:4.9)	0.80
Bicarbonate (mmol/L)	*1*	27 (24:28)	*3*	26 (23:29)	0.75
Lactate (mmol/L)	*1*	1.2 (0.9:1.7)	*3*	1.3 (0.9:1.7)	0.27
Sodium (mmol/L)	*1*	141 (137:144)	*3*	140 (137:143)	0.17
Potassium (mmol/L)	*1*	4.2 (3.9:4.5)	*3*	4.0 (3.8:4.3)	0.02
Glucose (mmol/L)	*1*	8.3 (7.4:9.7)	*3*	8.8 (7.3:10.1)	0.49
CRP (mg/L)	*1*	126 (51:196)	2	123 (48:206)	0.67
Leukocytes (10^9^/L)	*0*	12.5 (10.1:14.5)	*1*	12.0 (8.3:16.0)	0.58
Platelets (10^9^/L)	*0*	279 (190:382)	*1*	234 (169:358)	0.21
PT (INR)	*15*	1.1 (1.0:1.2)	*70*	1.1 (1.0:1.2)	0.12
APTT (sec)	*16*	40 (34:101)	*70*	46 (38:101)	0.04
Fibrinogen	*28*	6.25 (4.38:6.98)	*107*	5.70 (4.25:7.12)	0.12
D‐dimer (mg/L)	*14*	11 (5.8:17)	*66*	3.6 (1.9:9.1)	0.10
Troponin T (ng/L) *before 2021*	*5*	17 (8.2:77)	*10*	38 (26:99)	0.76
Troponin I (ng/L) *from 2021*	*10*	18 (4.5: 53)	*53*	34 (9.1;119)	0.13
Creatinine (μmol/L) before CTPA	*0*	84 (62:120)	*2*	79 (61:121)	0.58
Creatinine (μmol/L) the day after CTPA	*1*	82 (64:145)	*5*	83 (61:124)	0.91
Creatinine (μmol/L) at ICU discharge	*2*	68 (53:101)	*6*	71 (52:108)	0.45

*Note:* Values given as median (Q1:Q3) with *p*‐values referring to the Mann–Whitney *U*‐test. Italic figures indicate missing data. Group numbers for Troponin indicated in the rows.

Abbreviations: APTT, activated partial thromboplastin time; CRP, C‐reactive protein; CTPA, computed tomography pulmonary angiography; INR, international normalized ratio; P_a_CO_2_, partial pressure of arterial carbon dioxide; P_a_O_2_, partial pressure of arterial oxygen; PT, prothrombin time.

**TABLE 3:** Vital signs and RV/LV ratio at the time of CTPA exam.

Variable	*Missing*	CTPA positive *n* = 52	*Missing*	CTPA negative *n* = 191	*p*
Body temperature (°C)	*0*	37.6 (37.1:38.2)	*2*	37.4 (36.6:38.1)	0.05
SaO_2_ (%)	*0*	97 (95:98)	*2*	97 (95:99)	0.95
FiO_2_	*1*	0.55 (0.40:0.65)	*4*	0.55 (0.40:0.65)	0.74
PaO_2_/FiO_2_ (kPa)	*1*	21.0 (15.8:26.7)	*5*	20.2 (15.0:29.5)	0.51
Heart rate (BPM)	*0*	78 (68:88)	*0*	80 (68:95)	0.49
Systolic BP (mmHg)	*0*	123 (110:148)	*2*	123 (108:142)	0.45
Diastolic BP (mmHg)	*0*	62 (56:66)	2	60 (53:68)	0.51
RV/LV ratio	*8*	0.89 (0.84:0.97)	*10*	0.86 (0.81:0.97)	0.22
RV/LV ratio > 0.9	*8*	10 (19)	*10*	68 (36)	0.39

*Note:* Values for continuous variables given as median (Q1:Q3) with *p*‐values referring to the Mann–Whitney *U*‐test. Values for dichotomous variables given as number (%) with *p*‐values referring to the Fischer's exact test. Italic figures indicate missing data.

Abbreviations: BP, blood pressure; BPM, beats per minute; CTPA, computed tomography pulmonary angiography; FiO_2_, fraction of inspired oxygen; PaO_2_, partial pressure of arterial oxygen; RV/LV, right to left ventricular transverse diameter ratio; SaO_2_, arterial hemoglobin oxygen saturation.

The reason for ordering a CTPA was not specified for 2 (3.8%) of the patients with and 32 (17%) of the patients without a positive exam. “Elevated D‐dimer” was the only reason being more prevalent among patients with a positive CTPA exam (Table 4). “Elevated D‐dimer” and “Undefined clinical suspicion” were significantly associated with a positive exam in the multivariable logistic regression (Table 5).

**TABLE 4:** Arbitrarily categorized reasons in the CTPA exam orders.

Reason	CTPA positive *n* = 52	CTPA negative *n* = 191	*p*
Hypoxemia	27 (52)	106 (55)	0.75
Hypercapnia	0	4 (2.1)	0.58
Unstable circulation	5 (9.6)	38 (20)	0.10
Elevated D‐Dimer	21 (40)	32 (17)	< 0.05
Undefined clinical suspicion	9 (17)	17 (8.9)	0.12

*Note:* Values given as number (%) with *p*‐values referring to the Fischer's exact test. There were no missing values. The sum of proportions may exceed 100% as more than one reason could be present in one patient order.

Abbreviations: CTPA, computed tomography pulmonary angiography; PE, pulmonary embolism.

**TABLE 5:** Association between arbitrary reasons in the CTPA order and a positive exam.

Reason	OR	95% CI	*p*
Hypoxemia	1.28	0.62–2.7	0.51
Hypercapnia	0.80	0.01–8.4	0.88
Unstable circulation	0.88	0.29–2.3	0.81
Elevated D‐Dimer	4.03	1.9–8.8	< 0.01
Undefined clinical suspicion	3.73	1.3–11	0.02

*Note:* Multivariable analysis using penalized logistic regression with Firth's correction. There were no missing values. *p* < 0.05 is considered significant.

Abbreviations: CI, confidence interval; CTPA, computed tomography pulmonary angiography; OR, odds ratio.

The selection of patient variables associated with a positive CTPA exam in the univariable logistic regression (Table S1) included male sex, diabetes Type 2, and surgery within 30 days, but none of these was significantly associated with a CTPA finding of PE in the multivariable analysis (Table 6).

**TABLE 6:** Association between selected patient variables and a positive CTPA exam.

Variable	OR	95% CI	*p*
Biometrics			
Male	2.06	0.96–4.8	0.08
Co‐morbidities			
Diabetes Type 2	1.87	0.88–3.9	0.09
ICU diagnosis at risk for VTE			
Surgery within 30 days	1.88	0.93–3.7	0.07

*Note:* Multivariable regression analysis with variables selected by an OR > 1, a *p* ≤ 0.20 in the univariable analysis and missing data < 5%. There were no missing values. *p* < 0.05 is considered significant.

Abbreviations: CI, confidence interval; CTPA, computed tomography pulmonary angiography; OR, odds ratio.

## 4 Discussion

In this study, we found a diagnostic yield of 21% [95% CI: 17–26] from 243 CTPA exams, performed due to suspicion of PE in mechanically ventilated, critically ill patients. In a recent report, including 2713 patients having undergone CTPA to rule out PE in a tertiary center, 54/352 (15%) patients referred from the ICU had a positive exam, but there was no information about the degree of PE suspicion or the content of the radiology orders [14]. Very few studies of PE in intensive care patients report the rate of positive CTPA exams according to pre‐test clinical suspicion, but we found one comparable study from a single ICU where all patients, requiring mechanical ventilation and a thoracic contrast‐enhanced CT for any reason, were subjected to radiographic analysis of PE signs. PE was diagnosed in 33 of 176 (19%) patients, of whom 20 were “silent” (not suspected) [15]. A key issue when interpreting the yield of a diagnostic test is whether it was applied to patients with specific indications, or whether all patients were screened regardless of symptoms and signs. In the referenced study with an overall CT positivity of 19%, the yield was 16% (20/127) among 127 referred without PE suspicion and 26% (13/49) among patients where PE was suspected [15]. Several other studies report a low CTPA diagnostic yield (2%–8%), having divided detected PE cases by the number of total ICU admissions [6, 13, 16, 17, 18], but these likely underestimate the yield among suspected cases. The true prevalence of PE occurring in patients during intensive care remains largely unknown, especially considering the high number of missed PE found in autopsy studies [19]. Studies of CTPA yield are more common in patients referred from emergency departments (EDs) or hospital wards on PE suspicion with results varying from small fractions to almost 35% depending on pretest probability requirements for ordering the exam [20, 21, 22, 23]. For the ED setting, the UK Royal College of Radiologists advises an acceptable level of positive CTPA yield to be between 15% and 37% [24]. Similar recommendations for patients referred from the ICU are lacking but the result of our study falls within that span.

We also found that, among the five categories of motivational reasons motivating the clinical PE suspicion in the CTPA orders, “Elevated D‐dimer” and “Undefined clinical suspicion” were predictors of a PE finding. These two reasons constituted 30% of all reasons identified, which may be interpreted as clinicians being less precise in predicting a positive PE exam from the patient characteristics they observed. To our knowledge, this question has not previously been investigated in the ICU setting. However, one study investigating the content of radiology orders from an ED also found that a high D‐dimer value, as a reason for ordering CTPA in suspicion of PE, was among the strongest predictors of a positive finding [22]. Although an increased D‐dimer level has a low specificity for PE in critically ill patients in general [25], it may strengthen the clinical picture when other potential signs of PE are present. However, although male sex, diabetes mellitus Type 2, and surgery within 30 days seemed important in the first univariable analysis, we did not find any of the selected patient variables significantly associated with a positive CTPA exam. This contrasts with a number of studies that have established similar patient properties and contextual factors purporting risk for developing VTE in the ICU [8, 26]. Contrarily to what may have been expected, a COVID‐19 diagnosis did not turn out to be a significant PE predictor in spite of the well described high incidence of VTE in these critically ill patients during the pandemic [27, 28]. This discrepancy from the literature was also observed for ICU diagnoses of trauma and TBI, which did not significantly predict a positive finding on CTPA [11, 29]. The lack of significant association between specific patient predictors and positive CTPA findings in our study may be explained by the limited number of patients in each diagnostic category.

The proportion of patients on pharmacological thromboprophylaxis did not differ between patients with and without a positive PE exam. The fact that more than 30% did not receive this treatment despite recommendations is of concern, albeit not uncommon in the literature [30]. Lastly, we found no difference between renal function before and after the CTPA exam. This agrees with a recent study of intensive care patients submitted to radiographical exams that included intravenous contrast media and the recently reported general low risk of contrast‐associated acute kidney injury [31, 32].

### 4.1 Limitations

This study has several limitations. We focused on sedated, mechanically ventilated patients to study a population where symptoms are concealed and physiological signs of PE are difficult to distinguish but that reduced the number of clinical patient variables available for analysis. The CTPA outcome should be regarded as robust, but the categorization of the reasons given in the CTPA orders was based on qualitative interpretation of free‐text in the referral orders which introduced observer bias and precluded imputation for missing information. The absence of standardized referral criteria and the frequent lack of physiologically defined indications in referral texts limit the precision and reproducibility of this categorization method. The retrospective design resulted in a number of missing values for variables such as coagulation lab tests, and we did not have access to possible dead‐space ventilation which may be important for suspecting undetected PE. The penalized logistic regression approach in the analysis of the order reasons was chosen due to sparse data and the absence of events for some of the reasons, which would have resulted in non‐estimable coefficients under standard logistic regression. However, we did not perform any imputation due to the skewed nature of the data. We did not have data to classify the PE risk according to the guidelines issued by the European Society of Cardiology, but the low RV/LV ratios indicated PE of less severe character, which limits the generalizability of the findings to unstable patients. Yet, our results support the well‐known need for better tools for structured assessment of the clinical risk of PE among patients in the ICU.

## 5 Conclusion

The diagnostic yield of clinical CTPA exams for suspected PE in mechanically ventilated patients fell within a range commonly cited for suspected acute PE in emergency department populations, but its association with clinical signs described in the CTPA order and with selected patient variables was low.

## Author Contributions

Erik Bruno: Acquired data, analyzed data and drafted the first version of the manuscript. Matilda Kewenter: Acquired and analyzed data. Angeliki Dimopoulou Creusen: Designed the work and assisted in the acquisition of data. Freyr Einarsson: Substantially revised the manuscript. Gaetano Perchiazzi: Substantially revised the manuscript. Kristina Svennerholm: Substantially revised the manuscript. Christian Rylander: Conceptualized the study and designed the work, interpreted the data and substantially revised the manuscript. All authors read and approved the final manuscript.

## Funding

This study was supported by departmental funding from the Uppsala University Hospital and the Uppsala University. Gaetano Perchiazzi and Christian Rylander are holders of the Alvar Gullstrand research grant (Gaetano Perchiazzi ALF‐938050; Christian Rylander ALF‐978293) but did not receive any specific grant from funding agencies in the public, commercial, or non‐profit sectors.

## Ethics Statement

The study was approved by the Swedish Ethical Review Authority (DNr 2022‐03568‐01). Patient consent was waived due to the retrospective nature of the analysis.

## Consent

The authors have nothing to report.

## Conflicts of Interest

Freyr Einarsson and Kristina Svennerholm declare having received lecture fees from Leo Pharma. All other authors declare no conflicts of interest.

## Supporting information


**Data S1:** aas70216‐sup‐0001‐Supinfo.docx.

**Table S1:** Association between single patient variables and a positive CTPA exam.

## Data Availability

Due to Swedish legislation, the database is not available to the public. Pseudonymized data can be shared upon reasonable request addressed to the corresponding author.

## References

[1] X. Gao , L. Zeng , H. Wang , et al., “Prevalence of Venous Thromboembolism in Intensive Care Units: A Meta‐Analysis,” Journal of Clinical Medicine 11, no. 22 (2022): 6691. 36431168 10.3390/jcm11226691 PMC9698016

[2] C. Minet , L. Potton , A. Bonadona , et al., “Venous Thromboembolism in the ICU: Main Characteristics, Diagnosis and Thromboprophylaxis,” Critical Care 19, no. 1 (2015): 287. 26283414 10.1186/s13054-015-1003-9 PMC4539929

[3] M. Remy‐Jardin , M. Pistolesi , L. R. Goodman , et al., “Management of Suspected Acute Pulmonary Embolism in the Era of CT Angiography: A Statement From the Fleischner Society,” Radiology 245, no. 2 (2007): 315–329. 17848685 10.1148/radiol.2452070397

[4] L. M. Bergman , M. E. Pettersson , W. P. Chaboyer , E. D. Carlström , and M. L. Ringdal , “Safety Hazards During Intrahospital Transport: A Prospective Observational Study,” Critical Care Medicine 45, no. 10 (2017): e1043–e1049. 28787292 10.1097/CCM.0000000000002653

[5] J. H. Rundback , D. Nahl , and V. Yoo , “Contrast‐Induced Nephropathy,” Journal of Vascular Surgery 54, no. 2 (2011): 575–579. 21741789 10.1016/j.jvs.2011.04.047

[6] D. Schramm , A. G. Bach , H. J. Meyer , and A. Surov , “Thrombotic Events as Incidental Finding on Computed Tomography in Intensive Care Unit Patients,” Thrombosis Research 141 (2016): 171–174. 27058274 10.1016/j.thromres.2016.03.030

[7] C. Katsios , M. Donadini , M. Meade , et al., “Prediction Scores Do Not Correlate With Clinically Adjudicated Categories of Pulmonary Embolism in Critically Ill Patients,” Canadian Respiratory Journal 21, no. 1 (2014): 36–42. 24083302 10.1155/2014/296161 PMC3938238

[8] C. S. Vrettou , E. Dima , and I. Sigala , “Pulmonary Embolism in Critically Ill Patients‐Prevention, Diagnosis, and Management,” Diagnostics (Basel) 14, no. 19 (2024): 2208. 39410612 10.3390/diagnostics14192208 PMC11475110

[9] J. P. Vandenbroucke , E. von Elm , D. G. Altman , et al., “Strengthening the Reporting of Observational Studies in Epidemiology (STROBE): Explanation and Elaboration,” International Journal of Surgery (London, England) 12, no. 12 (2014): 1500–1524. 25046751 10.1016/j.ijsu.2014.07.014

[10] W. H. Geerts , D. Bergqvist , G. F. Pineo , et al., “Prevention of Venous Thromboembolism: American College of Chest Physicians Evidence‐Based Clinical Practice Guidelines (8th Edition),” Chest 133, no. 6 (2008): 381S–453S. 18574271 10.1378/chest.08-0656

[11] C. S. Vrettou , E. Dima , N. R. Karela , I. Sigala , and S. Korfias , “Severe Traumatic Brain Injury and Pulmonary Embolism: Risks, Prevention, Diagnosis and Management,” Journal of Clinical Medicine 13, no. 15 (2024): 4527. 39124793 10.3390/jcm13154527 PMC11313609

[12] F. A. Klok , M. Kruip , N. J. M. van der Meer , et al., “Confirmation of the High Cumulative Incidence of Thrombotic Complications in Critically Ill ICU Patients With COVID‐19: An Updated Analysis,” Thrombosis Research 191 (2020): 148–150. 32381264 10.1016/j.thromres.2020.04.041 PMC7192101

[13] J. G. Muscedere , D. K. Heyland , and D. Cook , “Venous Thromboembolism in Critical Illness in a Community Intensive Care Unit,” Journal of Critical Care 22, no. 4 (2007): 285–289. 18086398 10.1016/j.jcrc.2007.02.003

[14] T. Aggarwal , A. Eskandari , S. Priya , et al., “Pulmonary Embolism Rule Out: Positivity and Factors Affecting the Yield of CT Angiography,” Postgraduate Medical Journal 96, no. 1140 (2020): 594–599. 31907225 10.1136/postgradmedj-2019-137031

[15] C. Minet , M. Lugosi , P. Y. Savoye , et al., “Pulmonary Embolism in Mechanically Ventilated Patients Requiring Computed Tomography: Prevalence, Risk Factors, and Outcome,” Critical Care Medicine 40, no. 12 (2012): 3202–3208. 23164766 10.1097/CCM.0b013e318265e461

[16] S. Beitland , H. Wimmer , T. Lorentsen , et al., “Venous Thromboembolism in the Critically Ill: A Prospective Observational Study of Occurrence, Risk Factors and Outcome,” Acta Anaesthesiologica Scandinavica 63, no. 5 (2019): 630–638. 30623406 10.1111/aas.13316

[17] R. J. Eck , L. Hulshof , R. Wiersema , et al., “Incidence, Prognostic Factors, and Outcomes of Venous Thromboembolism in Critically Ill Patients: Data From Two Prospective Cohort Studies,” Critical Care 25, no. 1 (2021): 27. 33436012 10.1186/s13054-021-03457-0 PMC7801861

[18] C. B. Huang , C. X. Hong , T. H. Xu , et al., “Risk Factors for Pulmonary Embolism in ICU Patients: A Retrospective Cohort Study From the MIMIC‐III Database,” Clinical and Applied Thrombosis/Hemostasis 28 (2022): 10760296211073925. 35043708 10.1177/10760296211073925 PMC8796081

[19] B. Marcoen , K. H. Blot , D. Vogelaers , and S. Blot , “Clinical vs. Autopsy Diagnostic Discrepancies in the Intensive Care Unit: A Systematic Review and Meta‐Analysis of Autopsy Series,” Intensive Care Medicine 50 (2024): 1971–1982. 39287650 10.1007/s00134-024-07641-y

[20] C. L. Low , R. Y. Kow , A. Abd Aziz , et al., “Diagnostic Yield of CT Pulmonary Angiogram in the Diagnosis of Pulmonary Embolism and Its Predictive Factors,” Cureus 15, no. 6 (2023): e40484. 37461753 10.7759/cureus.40484 PMC10349910

[21] D. Mountain , G. Keijzers , K. Chu , et al., “RESPECT‐ED: Rates of Pulmonary Emboli (PE) and Sub‐Segmental PE With Modern Computed Tomographic Pulmonary Angiograms in Emergency Departments: A Multi‐Center Observational Study Finds Significant Yield Variation, Uncorrelated With Use or Small PE Rates,” PLoS One 11, no. 12 (2016): e0166483. 27918576 10.1371/journal.pone.0166483 PMC5137866

[22] M. Rohacek , J. Buatsi , Z. Szucs‐Farkas , et al., “Ordering CT Pulmonary Angiography to Exclude Pulmonary Embolism: Defense Versus Evidence in the Emergency Room,” Intensive Care Medicine 38, no. 8 (2012): 1345–1351. 22584801 10.1007/s00134-012-2595-z

[23] S. K. Saini , Z. S. Khan , V. Do , and G. Keijzers , “Computed Tomography Pulmonary Angiogram Ordering, Adherence to Decision Rules and Yield in the Emergency Department: An Observational Study,” Emergency Medicine Australasia 36, no. 5 (2024): 726–731. 38698536 10.1111/1742-6723.14428

[24] R. M. Daoud , A. M. Mohamed , M. S. Almajthoob , et al., “Is CT Pulmonary Angiography Overutilized in the Evaluation of Patients With Suspected Pulmonary Embolism? A Retrospective Study,” Canadian Journal of Respiratory Therapy 61 (2025): 127660. 39822304 10.29390/001c.127660 PMC11735043

[25] M. Di Nisio , A. Squizzato , A. W. Rutjes , H. R. Buller , A. H. Zwinderman , and P. M. Bossuyt , “Diagnostic Accuracy of D‐Dimer Test for Exclusion of Venous Thromboembolism: A Systematic Review,” Journal of Thrombosis and Haemostasis 5, no. 2 (2007): 296–304. 17155963 10.1111/j.1538-7836.2007.02328.x

[26] M. Boddi and A. Peris , “Deep Vein Thrombosis in Intensive Care,” Advances in Experimental Medicine and Biology 906 (2017): 167–181. 27628009 10.1007/5584_2016_114

[27] F. A. Klok , M. Kruip , N. J. M. van der Meer , et al., “Incidence of Thrombotic Complications in Critically Ill ICU Patients With COVID‐19,” Thrombosis Research 191 (2020): 145–147. 32291094 10.1016/j.thromres.2020.04.013 PMC7146714

[28] A. Kollias , K. G. Kyriakoulis , S. Lagou , E. Kontopantelis , G. S. Stergiou , and K. Syrigos , “Venous Thromboembolism in COVID‐19: A Systematic Review and Meta‐Analysis,” Vascular Medicine 26, no. 4 (2021): 415–425. 33818197 10.1177/1358863X21995566 PMC8024143

[29] K. L. Cole , S. Nguyen , S. Gelhard , et al., “Factors Associated With Venous Thromboembolism Development in Patients With Traumatic Brain Injury,” Neurocritical Care 40, no. 2 (2024): 568–576. 37421493 10.1007/s12028-023-01780-8

[30] J. Helms , S. Middeldorp , and A. C. Spyropoulos , “Thromboprophylaxis in Critical Care,” Intensive Care Medicine 49, no. 1 (2023): 75–78. 36038712 10.1007/s00134-022-06850-7 PMC9422935

[31] F. Berglund , E. Eilertz , F. Nimmersjo , et al., “Acute and Long‐Term Renal Effects After Iodine Contrast Media‐Enhanced Computerised Tomography in the Critically Ill‐A Retrospective Bi‐Centre Cohort Study,” European Radiology 34, no. 3 (2024): 1736–1745. 37658144 10.1007/s00330-023-10059-7 PMC10873227

[32] M. R. Ehmann , J. Mitchell , S. Levin , et al., “Renal Outcomes Following Intravenous Contrast Administration in Patients With Acute Kidney Injury: A Multi‐Site Retrospective Propensity‐Adjusted Analysis,” Intensive Care Medicine 49, no. 2 (2023): 205–215. 36715705 10.1007/s00134-022-06966-w

