# Supplementary material for: Computed Tomography Yield and Predictors of Pulmonary Embolism in Mechanically Ventilated Patients—A Retrospective Study
Source: Acta Anaesthesiol Scand. 2026 Mar 8;70(4):e70216. doi: 10.1111/aas.70216 (PMC12967873; doi:10.1111/aas.70216)
Supplement: Supplementary file 2 — Table S1: Association between single patient variables and a positive CTPA exam. [file AAS-70-0-s001.docx]

**Table S1**

**Association between single patient variables and a positive CTPA exam**

| **Predictor** | ***missing*** | **OR** | **95% CI** | **p-value** |
| --- | --- | --- | --- | --- |
| **BIOMETRICS** |  |  |  |  |
| Age (years) | *0* | 0.98 | 0.96-0.99 | 0.03 |
| Male | *0* | 1.88 | 0.86-4.1 | 0.11 |
| BMI (kg/m2) | *11* | 1.01 | 0.96-1.1 | 0.79 |
| RV/LV ratio | *18* | 1.79 | 0.31-10 | 0.52 |
| RV/LV ratio >0.9 | *18* | 1.38 | 0.71-2.7 | 0.34 |
| **CO-MORBIDITIES** |  |  |  |  |
| Hypertension | *0* | 1.11 | 0.60-2.1 | 0.73 |
| Heart failure | *0* | 0.15 | 0.02-1.1 | 0.07 |
| Cardiac arrhythmia | *0* | 0.76 | 0.30-1.9 | 0.57 |
| Ischemic heart disease | *0* | 0.47 | 0.14-1.6 | 0.24 |
| COPD | *0* | N/A | N/A | N/A |
| Asthma | *0* | 0.11 | 0.01-0.8 | 0.03 |
| Restrictive lung disease | *0* | N/A | N/A | N/A |
| Diabetes type 1 | *0* | N/A | N/A | N/A |
| Diabetes type 2 | *0* | 1.64 | 0.80-3.4 | 0.17 |
| Chronic renal failure | *0* | N/A | N/A | N/A |
| Acute on chronic renal failure | *0* | N/A | N/A | N/A |
| Acute kidney injury | *0* | 0.72 | 0.20-2.6 | 0.61 |
| Coagulation disorder | *0* | 1.85 | 0.16-21 | 0.62 |
| Previous DVT | *0* | N/A | N/A | N/A |
| Previous PE | *0* | 0.73 | 0.08-6.4 | 0.78 |
| Malignancy | *0* | 0.54 | 0.22-1.4 | 0.20 |
| **ICU DIAGNOSIS AT RISK FOR PE** |  |  |  |  |
| Sepsis | *0* | 0.59 | 0.22-1.6 | 0.31 |
| Polytrauma | *0* | 1.40 | 0.36-5.5 | 0.63 |
| Isolated TBI | *0* | 1.43 | 0.53-3.9 | 0.48 |
| Surgery within 30 days | *0* | 1.67 | 0.85-3.3 | 0.13 |
| Covid-19 | *0* | 1.07 | 0.57-2.0 | 0.83 |
| **ICU STAY** |  |  |  |  |
| Neurosurgical ICU | *0* | 1.40 | 0.71-2.7 | 0.33 |
| Length of total ICU stay (per day) | *0* | 1.01 | 0.99-1.0 | 0.41 |
| Length of ICU stay before CT (per day) | *0* | 1.01 | 0.97-1.1 | 0.64 |
| **THROMBOPROPHYLAXIS** |  |  |  |  |
| Low Molecular Weight Heparin | *0* | 1.07 | 0.55-2.1 | 0.84 |
| **LAB DATA BEFORE CTPA EXAM** |  |  |  |  |
| Leukocytes (10^9^/L) | *2* | 1.02 | 0.97-1.1 | 0.50 |
| Platelets (10^9^/L) | *2* | 1.01 | 0.99-1.0 | 0.39 |
| PT (INR) | *85* | 0.14 | 0.13-1.5 | 0.10 |
| APTT (sec) | *87* | 0.95 | 0.91-1.0 | 0.08 |
| Fibrinogen (g/L) | *135* | 1.07 | 0.85-1.3 | 0.56 |
| D-dimer (mg/L) | *80* | 1.01 | 0.99-1.0 | 0.15 |
| Creatinine (μmol/L) | *2* | 1.00 | 0.99-1.0 | 0.71 |

Univariable logistic regression analysis. p ≤0.20 considered significant.
OR – Odds Ratio, CI – confidence interval. CTPA – Computed Tomography Pulmonary Angiography, BMI – Body Mass Index, COPD – Chronic Obstructive Pulmonary Disease, DVT – Deep Venous Thrombosis, PE – Pulmonary Embolism, VTE – Venous Thromboembolism, TBI – Traumatic Brain Injury, ICU – Intensive Care unit, PT – Prothrombin time, INR – International Normalized Ratio, APTT – Activated Partial Thromboplastin Time.
